# Geographic Expansion of Sporotrichosis, Brazil

**DOI:** 10.3201/eid2603.190803

**Published:** 2020-03

**Authors:** Isabella Dib Ferreira Gremião, Manoel Marques Evangelista Oliveira, Luisa Helena Monteiro de Miranda, Dayvison Francis Saraiva Freitas, Sandro Antonio Pereira

**Affiliations:** Oswaldo Cruz Foundation, Rio de Janeiro, Brazil

**Keywords:** Sporotrichosis, Sporothrix, mycosis, fungi, zoonoses, public health, One Health, Brazil

## Abstract

Brazil has experienced geographic expansion of zoonotic sporotrichosis. Social problems in the country contribute substantially to the expansion. A comprehensive sporotrichosis control program is beyond the sphere of public health. A One Health approach is needed to control the disease in animals and humans.

High rates of human cases of sporotrichosis caused by *Sporothrix brasiliensis* transmitted by cats have been reported in Brazil since 1998 (*1*). The main referral center for the treatment of this mycotic disease, Oswaldo Cruz Foundation (Fiocruz) in Rio de Janeiro, recorded ≈5,000 human cases during 1998–2015 (D.F.S. Freitas, unpub. data) and 5,113 feline cases during 1998–2018 (S.A. Pereira, unpub. data). However, these numbers only represent cases diagnosed at 1 institution, and actual incidence rates likely are higher.

During 1998–2017, Brazil experienced a geographic expansion of sporotrichosis. The southeast region had the largest occurrence of human and animal cases (*1*,*2*), but outbreaks and case reports of feline sporotrichosis have been described from other regions (*3*–*5*) (Figure). In regions only reporting feline cases, zoonotic transmission probably is going unnoticed.

**Figure:**
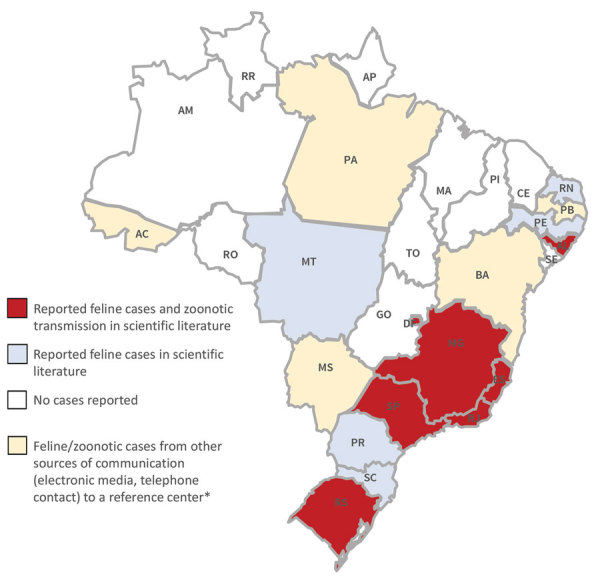
Occurrence of feline sporotrichosis and cases of zoonotic transmission in Brazil. *Reference center is the Laboratory of Clinical Research on Dermatozoonoses in Domestic Animals, Evandro Chagas National Institute of Infectious Diseases, Oswaldo Cruz Foundation (Fiocruz), Rio de Janeiro, Brazil. AC, Acre; AL, Alagoas; AM, Amazonas; AP, Amapá; BA, Bahia; CE, Ceará; DF, Federal District; ES, Espírito Santo; GO, Goiás; MA, Maranhão; MG, Minas Gerais; MS, Mato Grosso do Sul; MT, Mato Grosso; PA, Pará; PB, Paraíba; PE, Pernambuco; PI, Piauí; PR, Paraná; RJ, Rio de Janeiro; RN, Rio Grande do Norte; RO, Rondônia; RR, Roraima; RS, Rio Grande do Sui; SC, Santa Catarina; SE, Sergipe; SP, São Paulo; TO, Tocantins.

Zoonotic sporotrichosis also has been reported in the United States, India, Malaysia, Argentina, Mexico, and Panama (*2*). In Malaysia, isolates from cases caused by *S. schenckii* sensu stricto (*6*) have included clonal reproduction, which could indicate ongoing emergence of a genotype that is adapting to the feline host (*7*), similar to what was reported for *S. brasiliensis* in Brazil (*1*). Also, the occurrence of zoonotic sporotrichosis due to *S. brasiliensis* in Argentina is alarming because it points to a potential transboundary expansion of this virulent species to other regions in Latin America. Despite rules implemented for pet travel, poor control over road transportation might contribute to the spread of sporotrichosis in Brazil and could pose a risk for spread beyond its borders (*8*).

Fungal infections generally are neglected (*9*), and public health policies and strategic plans for prioritizing such infections are lacking. Inadequate surveillance of fungal infections leads to unnoticed emergence, such as seen with zoonotic sporotrichosis.

The rise and spread of sporotrichosis cases in Brazil were overlooked for several years, making a previously rare disease frequent and uncontrolled in many regions. Continuing socioeconomic and environmental difficulties, such as economic and social inequality, poverty, unemployment, urban agglomeration, and poor basic sanitation, coupled with scarce and inadequate health services, are fueling this expansion. In Rio de Janeiro, despite the high number of cases and the strain sporotrichosis puts on public health services, an animal sporotrichosis control program that included free diagnosis and treatment was not implemented until 16 years after the epidemic began. Nevertheless, given the chaotic situation in this region, the control measures used were insufficient. Even with the spread of the disease to other states in Brazil, compulsory notification is performed by only a few specific municipalities.

The absence of a comprehensive feline sporotrichosis control program in Brazil, the multifactorial difficulty in managing sick cats, and the lack of knowledge of sporotrichosis control measures by most of the population have contributed to the growing number of human and animal cases. A One Health approach is key for effective surveillance and successful control. Coordinated actions among veterinarians, laboratory practitioners, surveillance authorities, and other healthcare workers will ensure broader investigations and promote prevention, detection, and assistance for human and animal cases.

Early diagnosis of feline sporotrichosis is essential to guarantee appropriate prevention for owners, especially those at higher risk for infection, such as persons with immunosuppression. In addition, prompt treatment in felines can rapidly reduce the fungal load and risk for transmission of *Sporothrix* by cats (*10*). Thus, the availability of itraconazole, the first-line treatment for humans and animals, is essential in health units of affected areas. 

The pattern of feline sporotrichosis appears to be changing in the world, with new cases of zoonotic transmission by other *Sporothrix* species appearing (*1*). Health authorities from neighboring countries should be aware of the signs and symptoms of disease to identify cases early and rapidly implement prevention and control measures. Atypical cases and treatment failures emphasize the need for studies focusing on the detection of potential antifungal resistance and alternative therapeutic strategies. The emergence of new species or changes in the behavior of known species also should be assessed, to identify variations in the ecoepidemiology and in host–pathogen interactions.

If health authorities in Rio de Janeiro had taken measures to control and prevent sporotrichosis in the feline population at the first appearance of human cases, the current scenario could be different and likely would have cost less to the health system in the long term. Considering the remarkable spread of sporotrichosis in the past decade, effective public health actions, including free medication and service for animals, are urgently needed to prevent additional cases in affected areas. We encourage a One Health approach to curb further expansion of sporotrichosis in humans and animals in Brazil.
